# Pilot Scale Assessment of High-Pressure Processing (HPP) to Enhance Microbiological Quality and Shelf Life of Fresh Ready-to-Eat (RTE) Blue Crab Meat

**DOI:** 10.3390/microorganisms11122909

**Published:** 2023-12-02

**Authors:** Olivia Gilstrap, Chengchu Liu, Caleb Nindo, Salina Parveen

**Affiliations:** 1 Center for Food Science and Technology, Department of Agriculture, Food and Resource Sciences, University of Maryland Eastern Shore, Princess Anne, MD 21853, USA; oliviagilstrap@gmail.com (O.G.); sparveen@umes.edu (S.P.); 2 Maryland Sea Grant Extension Program, College of Agriculture and Natural Resources, University of Maryland, College Park, MD 21742, USA

**Keywords:** ready-to-eat (RTE) seafood, non-thermal processing, high-pressure processing (HPP), crab meat, food quality, shelf life extension

## Abstract

Blue crab (*Callinectes sapidus*) is a highly valuable wild fishery species of crab native to the waters of the western Atlantic Ocean and the Gulf of Mexico. The annual commercial production of live blue crabs is approximately 50,000 metric tons with a dockside value of USD 200 million. Presently the US blue crab processing industry sells crab meat in three basic forms: fresh crab meat, pasteurized crab meat, and frozen crab meat. By far “Fresh” is the most desirable form of crab meat. However, fresh crab meat has a limited shelf life. This study evaluated the effects of high-pressure processing (HPP) on enhancing the microbiological quality and shelf life of blue crab meat. Live blue crabs were pressure-cooked in a retort (≥115 °C for 4–6 min). The crab meat was handpicked, packed in plastic containers with seals, subjected to HPP treatment, and stored at 4 °C. Container integrity and water leakage issues were examined by observation in addition to weight comparison before and after HPP treatment; the shelf life of crab meat with and without HPP treatments was examined via microbiological tests and sensory evaluations. Results show that polypropylene containers sealed with 10K OTR (oxygen transmission rate) film could withstand high pressure without water leakage issues; HPP treatment at 600 MPa for 3 min could extend the shelf life of fresh, cooked, and handpicked crab meat from 6 days to 18 days based on the strictest APC (aerobic plate account) limit (APC ≤ 100,000 CFU/g). The sensory quality of the HPP-treated crab meat was well accepted throughout the 3-week storage period. The results support the use of HPP as an effective non-thermal processing technology to enhance the microbiological quality and extend the shelf life of fresh RTE blue crab meat.

## 1. Introduction

Crab is the largest species by value harvested in the United States (US). The blue crab (*Callinectes sapidus*) is native to the western Atlantic Ocean and the Gulf of Mexico. It is the most valuable fish species in Chesapeake Bay, and it offers significant culinary and economic value in the US, particularly in the states of Maryland, Virginia, North Carolina, and Louisiana. According to the statistical data from Commercial Fisheries Landings of the (US) National Oceanic and Atmospheric Administration (NOAA), the commercial dockside volume of blue crab is approximately 50,000 metric tons each year with a dockside value of USD 200 million [1]. Presently, the US blue crab processing industry produces and sells three basic forms: fresh, frozen, and pasteurized.

Fresh crab meat is a perishable ready-to-eat product that is processed under very high quality and safety standards. Live crabs are cooked via pressurized steam, and the meat is handpicked and packed into containers, which are typically polyethylene or polypropylene snap-lid containers. The process for pasteurized crab meat involves exposing the fresh crab meat to mild heat processing in hermetic, anaerobic packaging (i.e., metal cans), whereas frozen crab meat will involve a quick-freezing step using liquid nitrogen and is generally packaged in plastic snap lid containers. Fresh crab meat usually has one week of shelf life under refrigeration, frozen crab meat has a 6–8-month shelf life under frozen storage, and pasteurized crab meat has an 8–18-month shelf life under refrigeration. Both cryogenic freezing and pasteurization processing can greatly extend the shelf life of blue crab meat compared to when it is in its fresh state [2]. However, they also have some drawbacks. While frozen crab meat can be similar to fresh when properly thawed, it can still have some shortcomings such as freezer burn, textural changes due to defrosting during transportation, excessive exudate during thawing, or just a general loss of flavor when frozen. Additionally, for both frozen and pasteurized crab meat, there can be issues of induced lipid oxidation, dehydration, loss of juiciness, and color [3]. The majority of blue crabs, about 80 to 90%, are sold as fresh, with frozen and pasteurized crab meat being produced to provide crab meat during the off-season when fresh crab meat is not available or in the case where it is specifically requested from buyers. By far “Fresh” is the most desirable form of domestic blue crab meat, with most consumers only taking the other two forms when fresh is not available; however, it is greatly limited by its short shelf life.

High-pressure processing (HPP) is also referred to as high hydrostatic pressure or ultra-high-pressure processing. HPP is a non-thermal process that subjects foods (either solid or liquid products) to a pressure of 100–600 Megapascal (MPa) using a liquid as the pressure transfer medium [4]. The use of cold water as a pressure medium induces minimal changes in sensory and nutritional properties and helps the development of clean-label seafood products. It has shown a lot of promise in the food industry due to its microbial inactivation capabilities. Usually, the application of HPP (400–600 MPa) can destroy common seafood pathogen cells, such as *Vibrio* and *Listeria* spp., and slow the growth of spoilage microorganisms [5]. The major advantage of HPP is that it inactivates bacteria and viruses without appreciably affecting sensory properties and nutritional constituents [6]. One study showed that high-pressure processing could extend the shelf life of salmon, cod, and mackerel by approximately 1–15 days [7]. In addition, reports show that HPP can be applied in a variety of seafood including fish [7,8,9,10,11,12], fish soup [13], squid [14,15,16], oysters, and other shellfish [17,18,19,20,21,22]. The HPP technology at a lower pressure (200–350 MPa) is also used to shuck shellfish (i.e., oysters, lobsters, crabs, mussels, clams, and scallops) to increase recovery of the edible meat [5]. Specifically for crab meat, several studies have explored HPP technology as well. One study investigated pressure-induced germination and inactivation of *Bacillus cereus* spores and their survival in fresh blue crab meat during storage [23]. Other studies explored the effects of HPP on protein fraction and gelation capacities of crab meat [24,25,26]. Additionally, the interactions among HPP and quality characteristics of shucked crab meat [27], uncooked ready-to-heat or pasteurized ready-to-eat crab claw meat products [28], and polyphenol oxidase, melanosis, and quality in ready-to-eat crabs during storage [29] have been evaluated. However, limited information is accessible to the crab meat industry for applying HPP to enhance the microbiological quality and shelf life of pre-cooked fresh blue crab meat. This study first tested different containers and seals to identify the optimal packaging of crab meat for HPP treatment, and then it evaluated the effects of HPP on the microbiological quality and shelf life of pre-cooked fresh blue crab meat in a pilot scale using commercially available materials and industry facilities.

## 2. Materials and Methods

### 2.1. Crab Cooking and Fresh Crab Meat Preparation

Fresh crab meat was purchased from a local commercial crab processing plant (Cambridge, MD, USA) that is FDA-registered. The plant operates under the FDA’s seafood HACCP regulation and is inspected regularly by the state health department and/or FDA. Briefly, live blue crabs (wild caught locally) were steamed in a pressured retort (≥115 °C for 4–6 min). The crab meat was handpicked, packaged into polypropylene containers, and weighed and sealed with lids (1 pound or 16 ounces or 452 g per container). The packaged crab meat was kept surrounded by ice in waxed cardboard seafood boxes and stored in a walk-in cooler overnight. The following day the chilled fresh crab meat was transported by a refrigerated truck to a local HPP toll processor for HPP treatment and transported by ice chest coolers to a local microbiological laboratory for further tests.

### 2.2. Crab Meat Packaging Selection

After consultations with local crab meat processors, HPP equipment manufacturers, and packaging companies, four different types of packaging (S1, S2, S3, and S4) were selected for this study. Each packaging consisted of a unique combination of a polypropylene container and seal with or without applying film, as shown in Table 1.

The S1, S2, and S3 containers and lids were kindly donated by Berry Global Group, Inc. (Evansville, IN, USA); the S4 container, lid, and shrink film by J.M. Clayton Seafood Company (Cambridge, MD, USA), and the 10K OTR film (10,000 Oxygen Transmission Rate) by the Multivac Group (Kansas, MO, USA). All lids were applied by hand. The film was sealed using package equipment, a Multivac T200, with customized tooling designed and manufactured to fit the Multivac T200 (Kansas, MO, USA) prior to applying the lid. A seal time of 2 s at 190 °C was used for all the containers.

### 2.3. HPP Treatment

The fresh crab meat packaged in different containers with lids (S1, S2, S3, and S4) was subjected to HPP treatment at 600 MPa for 3 min at 4 °C. HPP treatment was conducted in a commercial toll processing unit (Maryland Packaging, Elkridge, MD, USA) using a JBT Avure AV-10 HPP machine (vessel dimensions: 306 mm diameter and 1420 mm internal length; vessel capacity: 1290 lbs. per hour for 3 min hold; vessel volume: 100 L; water input temperature: 4 to 29 °C). The operational parameters for HPP treatment (combination of pressure–time–temperature) used in this study (600 MPa for 3 min at 4 °C) were previously selected and verified based on recommendations by the toll processor and the equipment manufacturer’s specifications. After HPP treatment, the products were immediately covered by ice, transported under refrigerated conditions, and stored in a walk-in cooler or refrigerator until used for various tests.

### 2.4. Package Integrity Examination

A package integrity examination was conducted during the 1st HPP trial. A total of 28 pounds (12.7 kg) of fresh crab meat were packed and sealed in four different types of packaging, S1, S2, S3, and S4 (7 containers for each type of packaging; 1 pound (482 g) of crab meat per container). Container integrity and water leakage issues were examined by a visual check along with weight comparison before and after HPP treatment. The crab meat was weighed and visually examined for any physical deformations (i.e., dents, warps, perforations, seal breakage, etc.) before and after HPP treatment to determine if the containers could withstand HPP treatment. The gross weight of each container including crab meat was 482 g (17oz) before HPP treatment (GW_Before_). After HPP treatment, the containers of crab meat were weighed a second time (GW_After_) to determine if the pressure transmitting fluid (water in this case) had entered the crab meat product during HPP treatment. The weight increase was calculated using the following equation:

Weight Increase (%) = (GW_After_ − GW_Before_) × 100/GW_Before_ (1)

Weight increase represents the amount of water being pushed into crab meat product by pressure during the HPP process. The package with no weight increase or the least weight increase after HPP treatment determined which form of packaging would be selected for the 2nd HPP trial.

### 2.5. Examination of Microbiological Quality of HPP Crab Meat

Microbiological quality and shelf life analysis were conducted during the 2nd HPP trial. A total of 200 S1 containers were randomly picked and packed with fresh crab meat (1 pound (482 g) per container) before being subjected to HPP treatment (600 MPa, 3 min, 4 °C) as described earlier. The control (fresh crab meat without HPP treatment) and HPP product were completely surrounded by ice and transported to a microbiological laboratory for testing total bacteria count or aerobic plate count (APC), lactic acid bacteria, yeast and mold, and coliform and *Escherichia coli* (*E. coli*).

The tests took place over 14 days for the control samples and 42 days for the HPP crab meat samples, with intermediate test points throughout the study. The test intervals for the control sample were days 0, 3, 6, 8, 10, 12, and 14. The test intervals for the HPP sample were days 0, 7, 14, 18, 22, 26, 30, 34, 38, and 42. At each test interval, 2 separate, unopened packages of the control or HPP product were used as duplicates. All tests (Table 2) were conducted according to the official methods approved by the Association of Official Agricultural Chemists (AOAC) (see Table 2 for a brief summary of all test procedures). All test results were expressed as averaged values of CFU/g or Log_10_ CFU/g in each test point where duplicates were used.

### 2.6. Evaluation of Organoleptic Acceptance of HPP Crab Meat

Organoleptic acceptance of HPP crab meat was evaluated through voluntary sensory surveys (IRBNet Package: 1933295-2). Fourteen participants were recruited to evaluate the appearance, smell, texture, and flavor of HPP crab meat. The participants were frequent crab meat consumers who knew how to evaluate the sensory quality of fresh crab meat. The evaluation occurred at the participant’s plant or home. All crab meat was iced and kept under refrigeration (≤4 °C or 40 °F) at the commercial crab meat processor’s walk-in cooler to ensure uniform temperature control before distribution to participants. Each participant received a total of 3 containers of HPP-treated crab meat to be held at refrigerated temperatures (≤4 °C, never frozen). They were asked to evaluate one container per week for 3 weeks and to record the results on a provided form. When opening and evaluating the crab meat, participants were asked to first rate the appearance, smell, texture, flavor, and overall taste of the fresh crab meat using a 7-point scale, where 1 = Completely Unacceptable, 2 = Very Poor, 3 = Poor, 4 = Fair, 5 = Good, 6 = Very Good, and 7 = Excellent. If the participants decided the crab meat was acceptable, they were asked to use the crab meat as a prime ingredient to make crab cakes using a suggested recipe or any recipe of their choice and record how it performed in comparison to the fresh crab meat they routinely used in their business or at home. If the products had signs of spoilage or were deemed not fit for consumption, the participants only evaluated and recorded the crab meat ratings based on appearance and smell before discarding the product.

### 2.7. Statistical Analysis

Welch’s *t*-test was used to analyze the data, based on the assumption of unequal variances. The determination of significance was based on a *p*-value of 0.05.

## 3. Results

### 3.1. Effect of HPP Treatment on Container Integrity

**Figure 1:**
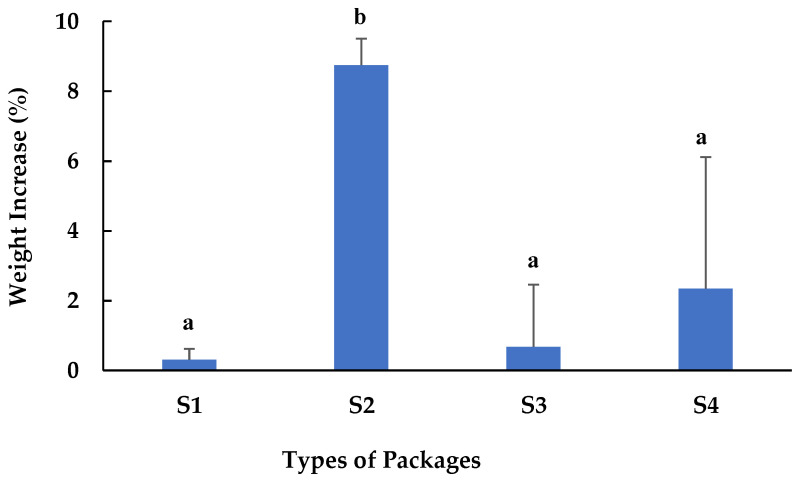
Amount of water (%) ingress into crab meat packed in different containers. Different letters above the bars indicate a significant difference (*p* ≤ 0.05).

A total of twenty-eight containers of pre-packaged fresh crab meat (seven containers for each package) were subjected to HPP treatment at 600 MPa for 3 min at 4 °C. After HPP treatment, all containers had no physical damage or deformations, but one of the S4 containers exploded during the HPP process due to a seal defect. The remaining 27 containers did not show dents, warps, perforations, or seal breakage after HPP treatment. This confirmed that all containers with proper seals tested can withstand the high-pressure treatment. However, weight comparison before and after HPP treatment suggested S1 had the best performance in preventing water within the pressure chamber from getting into the crab meat product, followed by S3 and S4. S2 exhibited the worst performance, with a weight increase higher than 8.7% (Figure 1). Although there were no significant differences between S1, S3, and S4, a separate trial was conducted, and the results for S3 and S4 showed higher levels of water ingress into the crab meat. Therefore, S1 was selected and used for additional trials on a larger scale to evaluate the effects of HPP on the microbiological and sensory quality of fresh crab meat.

### 3.2. Microbiological Quality of HPP Crab Meat

**Figure 2:**
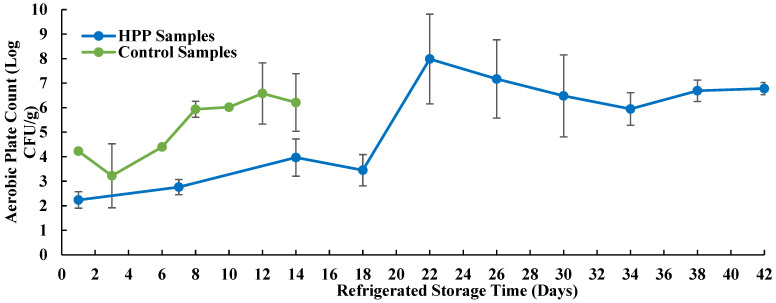
Growth levels of aerobic plate count during refrigerated storage (averaged values of duplicates expressed as CFU/g).

The control (fresh crab meat without HPP treatment) and HPP samples (fresh crab meat after HPP treatment) were tested for total aerobic bacteria count, sanitary indicator bacteria (coliform and *E. coli*), and spoilage microorganisms (lactic acid bacteria, yeast, and mold). The results are compared in Figure 2 and Table 3.

The aerobic plate count (Figure 2) for the control samples had a much higher starting result and continued to increase throughout the study. In comparison, the HPP samples had a much lower aerobic plate count at the beginning. The results remained relatively low until day 18 and showed a remarkable increase by day 22.

Both the control and HPP samples did not show any recovery of coliform or *E. coli* for the full study period (Table 3). Mold counts remained at lower counts with several test points until day 12 for the control and day 22 for the HPP samples, where counts showed an increase. Lactic acid bacteria and yeast demonstrated recovery from the control samples for most time points. These counts showed very slight increases throughout the study. For the HPP samples, lactic acid bacteria showed very slow growth until day 30; yeast counts showed much higher growth on day 14 and stayed high until day 30, where a drop in counts was observed. These highly variable counts between test points and sample containers can be attributed to homogeneity between packages or potentially competitive growth with other organisms, such as lactic acid bacteria and aerobic plate count.

### 3.3. Organoleptic Acceptance of HPP Crab Meat

**Figure 3:**
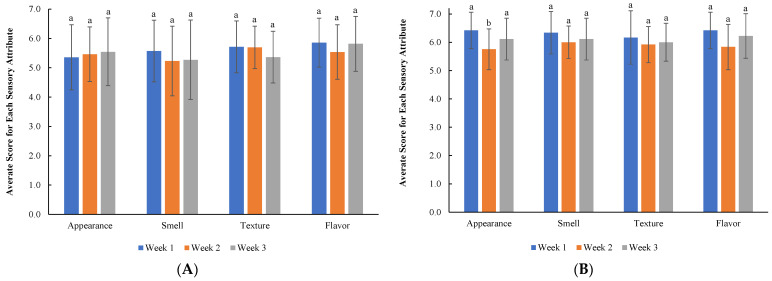
Averaged sensory scores of HPP crab meat (**A**) and crab cake (**B**). Different letters above the bars within each sensory attribute (appearance, smell, texture, flavor) indicate a significant difference (*p* ≤ 0.05) between weeks 1, 2, and 3.

A sensory survey was conducted to evaluate the organoleptic acceptability of HPP crab meat on appearance, smell, texture, flavor, and overall taste according to a 7-point scale as described earlier. For each sensory attribute, scores greater than 4 were considered acceptable, with 4 being borderline acceptable. Figure 3 shows the sensory survey results for each sensory attribute for crab meat (A) and crab cake (B). The average means of all sensory attributes of the crab meat were 6.3 ± 0.9, 5.2 ± 0.8, and 5.1 ± 1.0 at the end of week 1, week 2, and week 3, respectively; for crab cake, the average scores were 6.3 ± 0.6, 5.9 ± 0.5, and 6.1 ± 0.7 for week 1, week 2, and week 3, respectively. Therefore, participants rated the crab cake and plain crab meat similarly at week 1 and rated the crab cake slightly higher than the plain crab meat at weeks 2 and 3 for each test point. Statistical analysis showed that for both the crab meat and crab cake, there were no significant differences for all sensory attributes during the 3-week period of storage, except that the appearance of the crab cake in week 2 was significantly different from week 1. However, no significant difference was observed between week 1 and week 3. At the end of week 3, there was a commentary that the crab meat still smelt and tasted good, with no off flavors detected and a sweet taste still present.

## 4. Discussion

Fresh crab meat is one of the most perishable ready-to-eat (RTE) foods, and its spoilage is especially influenced by microbial growth, which can be accredited to some of crab meat’s intrinsic qualities, such as high-water activity and elevated nutrient content. Because of this, there is a present need for a way to limit the proliferation of these microorganisms and ultimately enhance the shelf life of blue crab meat [30]. HPP technology, at the ideal pressure, temperature, and hold time, has the capability to significantly reduce the microbial counts in foods, and when paired with good handling, processing, and storage practices, it can act as a key instrument in shelf life and quality enhancement. This study first evaluated the integrity of different containers against HPP treatment and then investigated the effectiveness of HPP treatment in enhancing the microbiological quality of fresh RTE crab meat packed in a selected container. Although physical-chemical parameters (such as pH, TVB-N, biogenic amines, and lipid oxidation) may also affect food product’s shelf life, our ongoing study showed that none of those parameters significantly changed during the whole period of storage (data not shown here). This was possibly because the crab meat was fully cooked and stored in refrigerated conditions for a relatively short period of time (up to 4 weeks). Therefore, the shelf life assessment in this study focused on microbiological quality and organoleptic acceptance after consultation with an ISO-accredited shelf life testing lab.

In the US, many blue crab processors use polypropylene (PP) containers to pack fresh, cooked, and handpicked crab meat. For HPP treatment to be successful, crab meat must be properly packaged and sealed to withstand high pressure and prevent water leakage. In the first trial of this study, four types of widely used PP cups were tested to evaluate container integrity (visual seal assessment and weight changes) against HPP treatment (600 MPa, 3 min). For HPP, the packaging used must be flexible and be able to withstand the compression and decompression cycles during the process. This allows the integrity of the packaging to be maintained, irreversible deformities to be avoided, and most importantly, it prevents the food product from leaking into the pressure vessel and the pressurizing liquid from infiltrating the package and mixing with the food product [31]. Visual examinations before and after HPP treatment showed that all tested crab meat containers could withstand the stresses of the HPP process. The absence of warping in all containers indicated that there were no issues with headspace because all of the tested containers were filled to the brim according to industry standards, which is ideal as headspace should be kept minimal to avoid excessive tensions on the packaging. For S1 cups, the film strength and seal were also visually assessed, as it is important that film seals are maintained and able to withstand the stresses of processing, including resistance to tears or perforations during the subsequent handling, distribution, and storage.

Weighing the crab meat containers before and after the HPP process was performed to assess any ingress of water from the vessel into the containers. As shown in Figure 1, the S1 containers had the least weight change (0.29% increase) compared to the other weight increases at 8.82%, 0.65%, and 2.35% for samples 2, 3, and 4, respectively. It is noted that the changes in weights for each type of container evaluated were minimal (all less than 57 g). These minimal differences could be due to a combination of the type of seal the various cups had and their instant reaction to the HPP process. This is further proof that each type of packaging evaluated was adequately sealed before being pressurized. If there were even the slightest openings in a seal before HPP treatment, the high hydrostatic pressure would have caused the water to enter the package, resulting in a much larger increase in weight or even an explosion of packaging, as was seen in one of the S4 cups. Therefore, the weight gain exhibited could potentially be due to the seal’s reaction during the decompression stage of HPP treatment, as the release of the pressure could have affected the seals in a way that allowed a minuscule amount of water to get into the containers.

Visual examinations before and after HPP treatment support the ability of all tested crab meat containers to withstand the stresses of the HPP process. Weighing the packaged crab meat before and after HPP provided insight into the various levels of seal strength among S1, S2, S3, and S4 types of packaging. Minimal to no weight differences are ideal, as they indicate the absence of water transferal into the crab meat. Therefore, S1 (packaging with 10K OTR film) was selected for moving forward with testing the microbiological and sensory quality of RTE fresh blue crab meat that would be treated under the same HPP parameters (600 MPa for 3 min). The 10K OTR film is a plastic film that has an oxygen transmission rate (OTR) of at least 10,000 cc/m^2^/24 h at 24 °C. It is a highly permeable film with water resistance and complies with FDA oxygen permeability guidelines for packaging fresh seafood to prevent *Clostridium botulinum* growth and toxin formation. The crab cooking process targets killing *Listeria monocytogenes*, but *Clostridium botulinum* may still survive. Therefore, fresh crab meat must be in packaging where there will be enough oxygen permeability. The other containers (S2, S3, S4) are considered oxygen permeable, so there is no concern for the growth of *Clostridium botulinum*.

In the second trial, HPP treatment at 600 MPa for 3 min was applied on RTE blue crab meat packaged in the selected S1 PP container with a 10K OTR film to investigate the effect of HPP treatment on fresh crab meat quality and shelf life. The microbiological quality of crab meat was determined through aerobic plate count (APC). A number of reports showed that certain acceptability thresholds were used to indicate food quality. For example, *Microbiological Guidelines for Food* [32] suggested that APC ≤ 5 Log CFU/g be considered as “Satisfactory”, APC between 5 Log and 7 Log CFU/g as “Borderline”, and ≥7 Log CFU/g as “Unsatisfactory” (for cooked foods chilled but with some handling prior to sale or consumption such as fresh crab meat). Others also reported that APC threshold values between five and seven log CFU/g indicate poor quality [33,34]. Specifically, in Maryland, the acceptable quality limit of APC for fresh blue crab meat is APC 100,000 CFU/g (5 Log CFU/g) [35]. In this study, the APC for control samples surpassed 5 Log CFU/g on day 8. Therefore, its shelf life was 6 days based on the 5 Log CFU/g limit. For the HPP samples, APC remained relatively low from the beginning to day 18 and showed a huge jump by day 22, where it also showed a greater than 5 Log CFU/g. Based on this, the HPP samples had at least 18 days of shelf life. APC 5 Log CFU/g is the strictest cutoff quality limit.

**Figure A1:**
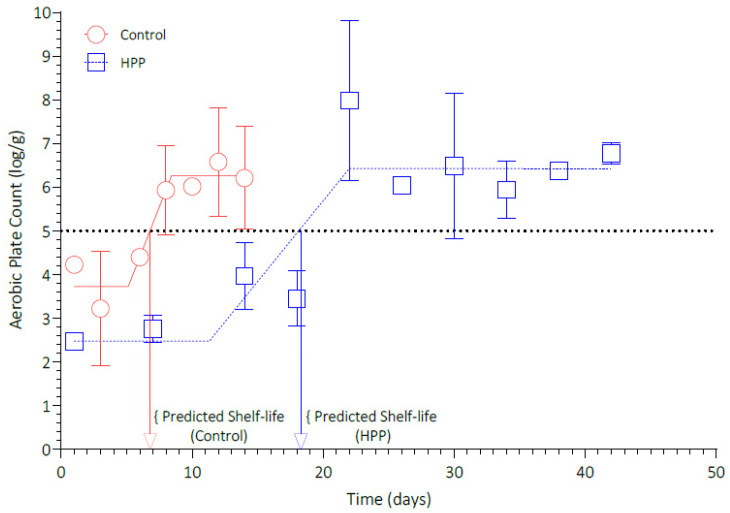
Change of aerobic plate count (APC) as a function of time for control crab meat and crab meat treated with hydrostatic high pressure (HPP). Data (symbols) were fitted to the three-phase linear model (lines). See text under Figure A1 for details.

It should be noted that there are no universal acceptability thresholds for APC since APC does not necessarily link to any unsafe food product or foodborne pathogen. As shown in Figure 2, the HPP samples had much lower APC at the beginning, and the results remained relatively low until day 18 but surpassed 5 Log CFU/g by day 22. Therefore, the HPP samples demonstrated a shelf life of at least 18 days. When the data were fitted to a three-phase linear growth model, a similar result was obtained (see Figure A1 in Appendix A for additional information). It seems that HPP treatment extends the shelf life of fresh crabs by increasing aerobic bacteria’s lag time and stationary phases and by reducing their growth rate (Figure A1).

The sensory evaluation results showed that the HPP crab meat was still acceptable on week 3, as shown in Figure 3. Starting at week 1, for sensory, the scores were mostly 5, 6, or 7, indicating good to excellent ratings. Commentary from participants was mostly positive and mentioned statements such as the crab meat having “no off flavor”, that “the meat looked and tasted good”, that it appeared “soft and delicate”, and that “it did not seem different from other crab meat”. Negative comments in week 1 mentioned that the crab meat had a more “yellowish rather than white color”, that it “looks too moist”, and that it “appeared squeezed/packed”. In week 2, the participants had similar comments in regard to “soft texture”, “no-off flavor”, and being “an excellent product”, with additional comments mentioning “the meat looked whiter with more flavor (sweetness) than the sample in previous week”, that the crab meat “still seems fresh”, and that they “noticed no change from the week before”. The few negative comments were still similar to week one, as previously mentioned. The third or final week’s comments and scores were also very similar to the previous weeks. Some particular week 3 comments mentioned that the crab meat was “much better than what was sold at Food Lion” and “no change from last week”, but there was a negative comment stating that the “texture may have seemed less fresh than the previous week”. However, it is worth noting that this participant still rated the crab meat texture a 4 for fair, with other attributes being rated at a 5 for good. In regard to the crab cakes, the reviews stayed fairly consistent from weeks 1 to 3. There were many comments in line with statements such as “very tasty”, “looks good, no off flavor”, a tender and soft texture”, and even at week 3 commenting that the crab cake was excellent and “would be acceptable for any use, either at home or in a fine dining restaurant”. The primary negative comments mentioned were from a couple of participants mentioning issues such as the presence of cartilage.

Overall, the crab meat received acceptable ratings throughout the entirety of the sensory study. Many of the issues mentioned, such as the yellow color of the meat and the presence of cartilage, were due to factors unrelated to the HPP process. The yellow color in crab meat is normal, as meat from female crabs can look yellow/orange in appearance, and the cartilage issue can be attributed to the handpicking skills of the employees at the crab processing facility. Points of concern that may be attributable to HPP treatment would primarily be in regard to comments about some containers being too moist, which could be the result of the water used in HPP treatment getting into some containers. Further consumer testing would be beneficial to confirm whether this would be a significant deterrent for consumers. It should also be noted that throughout the study, the number of negative remarks as the weeks progressed did not increase, but rather stayed relatively consistent each week, with some comments in weeks 2 and 3 actually expressing higher levels of liking than week 1. This further confirms the natural variation that can be expected in crab meat, as a new container of crab meat was provided to the participants to rate each week as opposed to having the same crab meat container for the participants to rate over the 3-week period. In regard to significant differences, there was only a significant difference between the crab cake appearance in week 2 versus week 1, as seen in Figure 3. Because the crab cake only had a significant difference and not the plain crab meat, this difference in rating was likely influenced by inconsistencies in how the crab cakes were prepared, as the participants took the crab meat home to taste and prepare the crab cakes. Still, both the plain crab meat and crab cake kept scores at 5 or higher up to week 3, denoting average ratings of good or very good, and even at week 3, there were still some participants who gave ratings of 7, meaning excellent.

In addition, the fresh crab meat was purchased from a local commercial crab processing plant (Maryland, USA) that is FDA-registered. Crabs are retorted in the shell. It is true that the fully cooked crabs are nearly sterile after the retort cooking process. However, crab meat is picked by hand. Microbial contamination or cross-contamination occurs during the handpicking process. Although the plant operates under the FDA’s seafood hazard analysis and critical control point (HACCP) regulation and is inspected regularly by the state health department and/or FDA, there was a lot of variability in the microbial counts among individual containers of crab meat directly picked from different crabs by different pickers. Since a new package of crab meat was opened on each test day, it is impossible to guarantee complete unison at the initial microbial levels for all the crab meat. Separate testing was conducted to evaluate the levels of variability in the microbial quality of crab meat, and the results support the aforementioned microbial variation (data not shown).

## 5. Conclusions

In summary, an HPP treatment at 600 MPa for 3 min was applied on fresh RTE blue crab meat in an industry-standard polypropylene cup sealed with a 10K OTR film. HPP treatment successfully extended the shelf life of fresh RTE blue crab meat from 6 days to 18 days using the strictest APC limit (APC ≤ 100,000 CFU/g). The sensory quality of the HPP-treated crab meat was well accepted through the whole 3-week storage period. These results demonstrate that HPP treatment can be applied in the blue crab industry as an effective practical technology to enhance the microbiological quality and shelf life of blue crab meat.

The crab meat industry is in need of a solution to address the ongoing issue of the short shelf life of fresh RTE crab meat, and due to this growing demand, the industry has supported this process and been involved throughout the course of this research. The industry is strictly upheld to the standards stipulated by the numerous Food and Drug Administration (FDA) seafood regulations designed to provide preventative measures for food safety hazards. These programs, such as HACCP, good manufacturing practices (GMPs), and Sanitation Standard Operation Procedures (SSOPs) have all been designed to work together to provide a comprehensive blue crab processing quality control program.

This study has been viewed by the blue crab industry as critical to its continued viability. The results demonstrated that HPP treatment can be applied in the blue crab industry as an effective non-thermal technology to enhance the microbiological quality and shelf life of fresh RTE blue crab meat. Since all HPP trials were conducted at a pilot scale in commercial facilities instead of at a lab scale, these results illustrate that HPP treatment, when applied at the parameters 600 MPa for 3 min on industry-standard polypropylene cups sealed with a 10K OTR film, can be incorporated into the seafood industry as a means to enhance the shelf life of crab meat. Adopting this advanced technology into its operation can provide the potential for a more competitive blue crab industry.

## Figures and Tables

**Table 1:** Selected packaging for testing packaging integrity against HPP treatment.

Selected Packaging	Container Code	Lid Type	Film Seal	Film Type
S1	T41016IMBCP	Standard single-seal lid	Yes	10K OTR * film
S2	T40616SLCP	Safe lock, tamper evident lid	No	N/A
S3	T41016CP	Recessed, tamper-resistant lid	No	N/A
S4	T41016CP	Standard single-seal lid	Yes	Shrink film

* The 10K OTR (oxygen transmission rate) film is a plastic film that has an oxygen transmission rate of at least 10,000 cc/m^2^/24 h at 24 °C. It is a highly permeable film with water resistance and complies with FDA oxygen permeability guidelines for packaging fresh seafood to prevent *Clostridium botulinum* growth and toxin formation.

**Table 2:** Test procedures for microbiological quality of crab meat.

Analysis	Method	Incubation Temperature/Time
Aerobic plate count	AOAC 990.12	35 °C/48 h
Total coliforms	AOAC 998.08	35 °C/48 h
*E. coli*	AOAC 998.08	35 °C/48 h
Lactic acid bacteria	AOAC PTM #041701	35 °C/48 h
Yeast	AOAC 997.02	25 °C/5 days
Mold	AOAC 997.02	25 °C/5 days

**Table 3:** Growth levels of sanitary indicator bacteria and spoilage bacteria in the HPP and control samples during refrigerated storage (individual values of duplicates expressed as CFU/g).

HPP Sample	Day 1	Day 7	Day 14	Day 18	Day 22	Day 26	Day 30	Day 34	Day 38	Day 42
Total coliforms	<10 *	<10	<10	<10	<10	<10	<10	<10	<10	<10
	<10	<10	<10	<10	<10	<10	<10	<10	<10	<10
*Escherichia coli*	<10	<10	<10	<10	<10	<10	<10	<10	<10	<10
	<10	<10	<10	<10	<10	<10	<10	<10	<10	<10
Lactic acid bacteria	10	<10	90	460	400	590	>300,000	<10	10	>300,000
	<10	<10	<10	<10	60	30	1300	50	9500	<10
Yeast	<10	30	<10	<10	>300,000	>300,000	<10	40	<10	>300,000
	<10	<10	560,000	55,000	<10	>300,000	<10	<10	<10	<10
Mold	<10	<10	<10	<10	3200	<10	<10	100	<10	>300,000
	<10	<10	10	<10	10	<10	<10	40	>300,000	<10
**Control Sample**	**Day 1**	**Day 3**	**Day 6**	**Day 8**	**Day 10**	**Day 12**	**Day 14**	ND **	**ND**	**ND**
Total coliforms	<10	<10	<10	<10	<10	<10	<10	ND	ND	ND
	<10	<10	<10	<10	<10	<10	ND	ND	ND	ND
*Escherichia coli*	<10	<10	<10	<10	<10	<10	ND	ND	ND	ND
	<10	<10	<10	<10	<10	<10	ND	ND	ND	ND
Lactic acid bacteria	90	<10	10	320	380	700	900	ND	ND	ND
	120	<10	10	110	660	680	100	ND	ND	ND
Yeast	<10	20	70	<10	<10	450	<10	ND	ND	ND
	<10	<10	<10	<10	<10	<10	300	ND	ND	ND
Mold	<10	<10	20	80	1000	20	30	ND	ND	ND
	<10	10	<10	350	<10	290	<10	ND	ND	ND

* <10: Below the detection limit; ** ND: did not test.

## Data Availability

All relevant data is contained within the article. The original contributions presented in the study are included in the article, further inquiries can be directed to the corresponding author.

## Appendix A

The aerobic plate count data for the Control and HPP-treated crabmeat during refrigerated storage were log10 transformed and then fited to the three-phase linear growth model [36] using GraphPad Prism (version 10, GraphPad Software Inc., San Diego, CA, USA). This was done to determine the effect of HPP on APC at day 1 of storage, lag time (LT), growth rate (GR), maximum population density (MPD), and time to MPD (tMPD). When possible, model parameters were compared for significant differences (*p* < 0.05) using the sums of squares F test in Prism.

Because of limited and variable data, goodness of fit of the model to the data was low for the Control and the HPP treatment. The coefficient of determination (R^2^) was 0.7178 for the Control and 0.7054 for the HPP treatment. The adjusted R^2^ was 0.6331 for the Control and 0.6374 for HPP treatment. The standard deviation of the residuals (Sy.x) was 0.8394 log/g for the Control and 1.195 log/g for the HPP treatment. The Root Mean Squared Error (RMSE) was 0.7362 for the Control and 1.077 for the HPP treatment. Also, LT and MPD parameters were unstable and thus, the 95% confidence intervals for all parameters were interpreted with caution.

Nonetheless, APC at Day 1 of storage was 3.73 log/g for the Control and 2.48 log/g for the HPP treatment. Because of missing data, a statistical comparison (i.e., sums of squares F test) of this parameter between the Control and HPP treatment could not be calculated.

Lag time was 5.1 days for the Control and 11.3 days for the HPP treatment. However, these values were not statistically different (*p* = 0.1255) because of variable and limited data.

Maximum population density was 6.27 log/g for the Control and 6.43 log/g for the HPP treatment. These values did not differ statistically (*p* = 0.6758).

Time to stationary phase (tMPD) was 8.4 days for the Control and 22 days for the HPP treatment. These values differed statistically (*p* = 0.0003).

The growth curves differed (*p* = 0.0003) between the Control and the HPP treatment.

Growth rate was 0.77 log/day for the Control and 0.37 log/day for the HPP treatment.

The interpolation function of GraphPad Prism was used to determine the time to reach 5 log/g (95% confidence interval) or the shelf-life of the crab meat. Shelf-life was 6.8 (95% CI = 5.1 to 8.4) days for the Control and 18.1 (95% CI = 11.3 to 22) days for the HPP treatment.

Thus, the HPP treatment lowered APC at Day 1 of refrigerated storage by 1.25 log/g, increased lag time by 6.2 days, decreased growth rate by 0.4 log/day, and increased time to 5 log/g or shelf-life by 11.3 days. However, because of limited and variable data, these trends were not statistically significant, and the model had low goodness-of-fit. Nonetheless, the results look promising and suggest that the decrease in growth rate by the HPP treatment could be from a change in the makeup of the microbial population of crab meat that could be further investigated using microbiomics.
